# Current Status of ChatGPT Use in Medical Education: Potentials, Challenges, and Strategies

**DOI:** 10.2196/57896

**Published:** 2024-08-28

**Authors:** Tianhui Xu, Huiting Weng, Fang Liu, Li Yang, Yuanyuan Luo, Ziwei Ding, Qin Wang

**Affiliations:** 1 Clinical Nursing Teaching and Research Section The Second Xiangya Hospital of Central South University Changsha China; 2 Xiangya School of Nursing Central South University Changsha China

**Keywords:** chat generative pretrained transformer, ChatGPT, artificial intelligence, medical education, natural language processing, clinical practice

## Abstract

ChatGPT, a generative pretrained transformer, has garnered global attention and sparked discussions since its introduction on November 30, 2022. However, it has generated controversy within the realms of medical education and scientific research. This paper examines the potential applications, limitations, and strategies for using ChatGPT. ChatGPT offers personalized learning support to medical students through its robust natural language generation capabilities, enabling it to furnish answers. Moreover, it has demonstrated significant use in simulating clinical scenarios, facilitating teaching and learning processes, and revitalizing medical education. Nonetheless, numerous challenges accompany these advancements. In the context of education, it is of paramount importance to prevent excessive reliance on ChatGPT and combat academic plagiarism. Likewise, in the field of medicine, it is vital to guarantee the timeliness, accuracy, and reliability of content generated by ChatGPT. Concurrently, ethical challenges and concerns regarding information security arise. In light of these challenges, this paper proposes targeted strategies for addressing them. First, the risk of overreliance on ChatGPT and academic plagiarism must be mitigated through ideological education, fostering comprehensive competencies, and implementing diverse evaluation criteria. The integration of contemporary pedagogical methodologies in conjunction with the use of ChatGPT serves to enhance the overall quality of medical education. To enhance the professionalism and reliability of the generated content, it is recommended to implement measures to optimize ChatGPT’s training data professionally and enhance the transparency of the generation process. This ensures that the generated content is aligned with the most recent standards of medical practice. Moreover, the enhancement of value alignment and the establishment of pertinent legislation or codes of practice address ethical concerns, including those pertaining to algorithmic discrimination, the allocation of medical responsibility, privacy, and security. In conclusion, while ChatGPT presents significant potential in medical education, it also encounters various challenges. Through comprehensive research and the implementation of suitable strategies, it is anticipated that ChatGPT’s positive impact on medical education will be harnessed, laying the groundwork for advancing the discipline and fostering the development of high-caliber medical professionals.

## Introduction

Artificial intelligence (AI) is the simulation of human cognitive capacities using computer programming, allowing robots to emulate human thought and behavior [1]. AI generation is the automated creation of various content formats, such as text, photos, video, and audio, using AI technologies. This approach generates content using language, visuals, and multimodal macro models [2]. Among these technologies, ChatGPT stands out as a sophisticated, large-scale language model developed by OpenAI, which has reached stage 4.0, the most recent iteration of the OpenAI system. ChatGPT, which has been trained on large amounts of textual data, is designed to participate in conversational exchanges with users while responding contextually to their prompts [3]. Particularly, ChatGPT has advanced capabilities in natural language processing, logical reasoning, task execution, information retrieval, picture analysis, content development, and other areas [4]. Moreover, ChatGPT provides a vast range of services accessible through global registration, facilitating its integration across various domains.

In the field of education, the introduction of ChatGPT has generated considerable interest and prompted in-depth discussions. Its exceptional generating capabilities offer new avenues for scholarly research, increased learning, classroom improvement, and knowledge sharing [5]. The incorporation of ChatGPT into medical education has become a major focus, with the goal of combining the advancements of education and medicine. In order to provide a well-informed assessment of the potential and limitations of integrating ChatGPT into medical education, this research aims to examine both the technology’s capabilities and the challenges it may present. Furthermore, tactics are put out to support the smooth integration of technology and medicine.

## Status of ChatGPT in Medical Education

**Figure 1:**
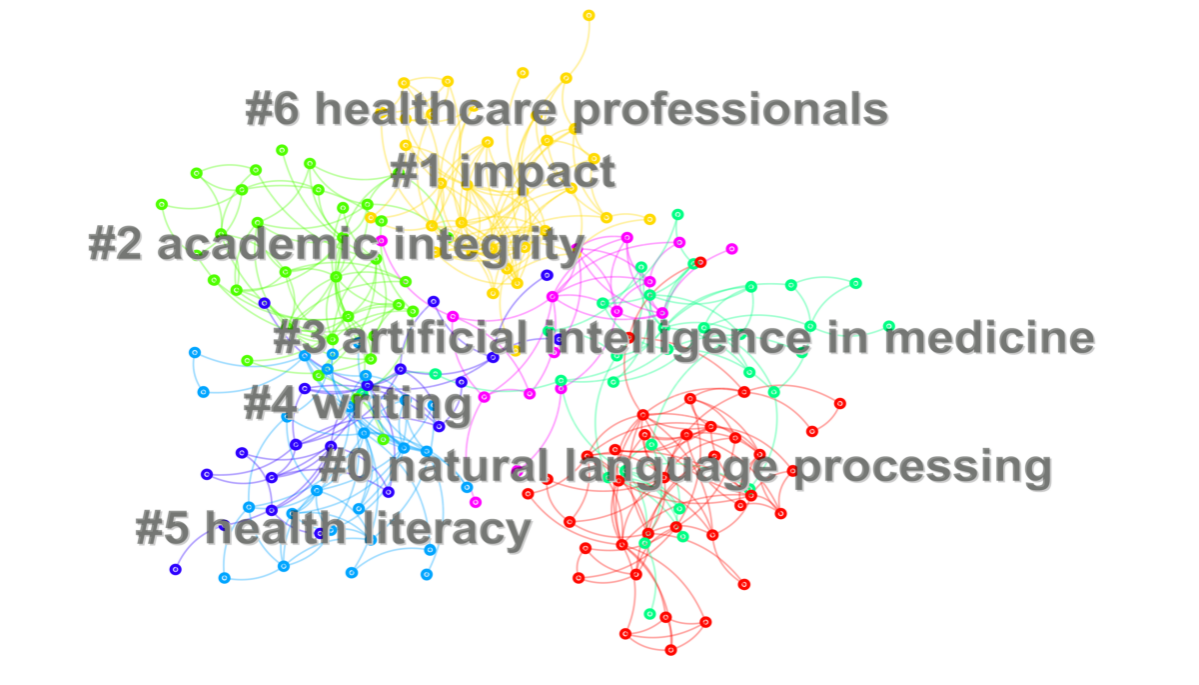
Keywords of ChatGPT in medicine and education.

**Figure 2:**
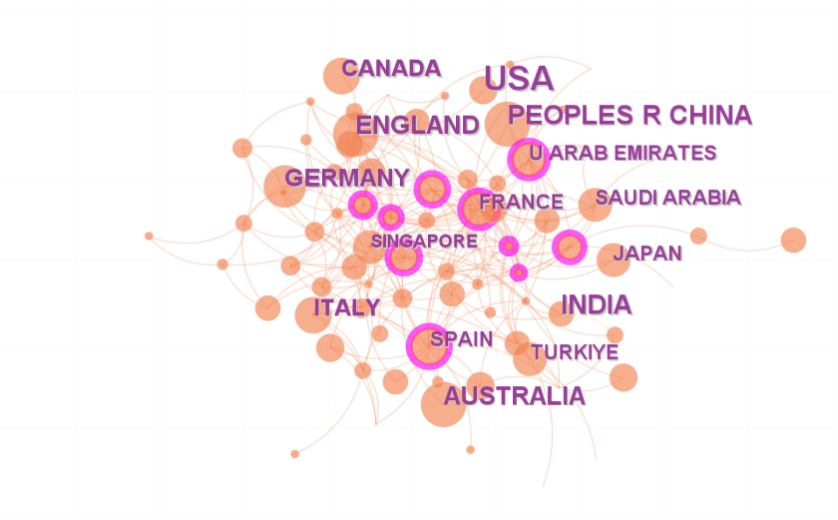
National publications of ChatGPT in medicine and education.

In this study, we searched the Web of Science database using specific terms related to ChatGPT and education or medical science within the timeframe of January 2022 to December 2024. The search formula is (“Chat generative pretrained transformer” OR “chat GPT” OR ChatGPT OR chat- GPT OR GPT-3.5 OR GPT-4.0”) AND (Education OR educate OR educator OR Students, Medical OR medical science OR medicine OR health care OR health science). We used CiteSpace v6.1R6 (64-bit) Basic to analyze the posted keywords [6]. The basic parameters used in CiteSpace were configured as follows: the time partition ranged from January 2022 to December 2024, with a one-time slice; the node selection criterion was set to k=25 for the g-index in each time slice, while the remaining parameters were kept at default settings. The top 7 keywords, ranked by frequency in the keyword network, are natural language processing, impact, academic integrity, AI in medicine, writing, health literacy, and health care professionals (Figure 1), and the top 10 countries by publication volume are United States, India, China, Australia, England, Germany, Canada, Italy, Spain, and United Arab Emirates (Figure 2).

Next, we will analyze the impact of ChatGPT and focus on its advantages, disadvantages, and coping strategies in the areas of education, academics, clinical decision-making, and health education.

## Potentials

**Figure 3:**
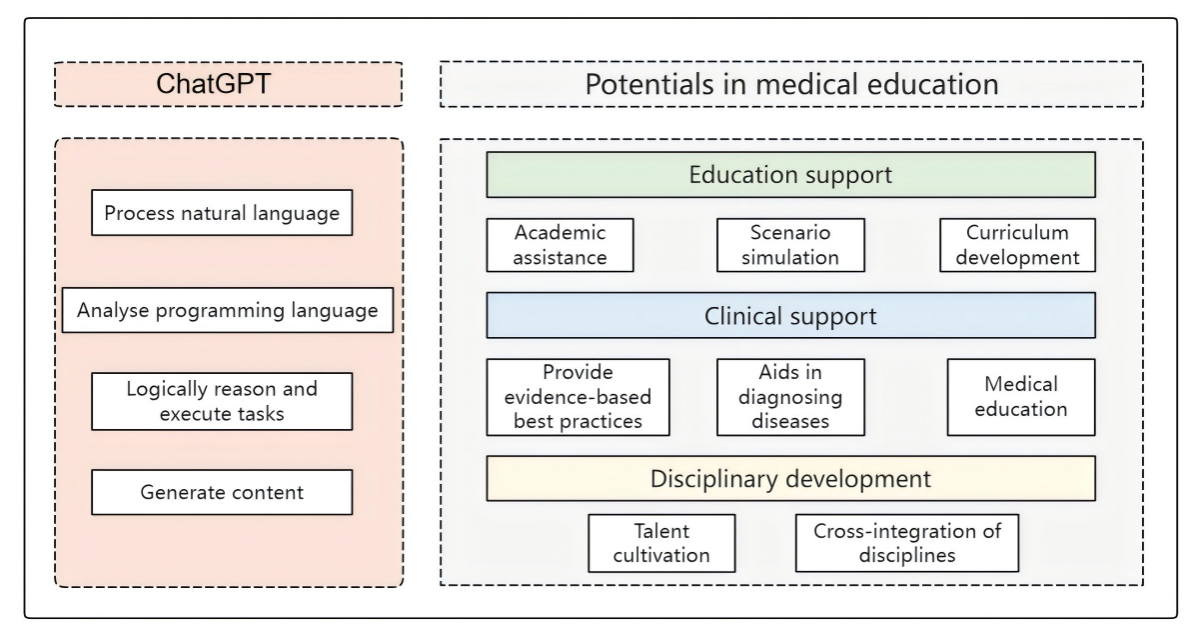
Functions of ChatGPT and the potentials of medical education.

A summary of the functions of ChatGPT and its potential role in the field of medical education is shown in Figure 3.

### Education Support

#### Academic Assistance

ChatGPT excels in both language understanding and content generation. It relies on semantic understanding and reasoning to decipher user intent through mutual dialog. In addition, by applying deep learning techniques, ChatGPT efficiently retrieves information from a variety of sources to provide consumers with reliable answers [7]. As a result, ChatGPT emerges as an invaluable resource for medical students, especially in helping them understand complex ideas. ChatGPT improves topic understanding by providing examples and conducting text analyses [8]. Furthermore, ChatGPT serves an important role in alleviating academic difficulty among medical students [9]. With its ability to produce and answer questions as well as assist with revision tasks, ChatGPT can help medical students complete coursework, assess the quality of their coursework, and reinforce previously learned concepts [10]. Serving as a personalized tutor, it develops customized learning programs and time management strategies based on individual interests and learning preferences [11]. In addition, renowned for its prowess in research and writing, ChatGPT contributes significantly to academic endeavors [12]. On one hand, it assists students in comprehensively exploring research literature, gaining a preliminary understanding of current research trends [13]. On the other hand, ChatGPT aids in structuring thesis frameworks and generating writing prompts, while also offering grammar and spelling checks to enhance writing proficiency and quality [14].

#### Scenario Simulation

ChatGPT has superior social features that allow it to replicate clinical settings for medical students through situational simulation and role-playing [15]. This functionality assists the transition of medical students from a theoretical to a clinical attitude. Furthermore, ChatGPT provides the potential to faithfully mimic clinical circumstances while dynamically adapting to changes in patients’ conditions [16]. This function lets students gain practical experience managing unexpected medical problems in simulated contexts, thereby improving their preparedness and psychological fortitude.

#### Curriculum Development

**Figure 4:**
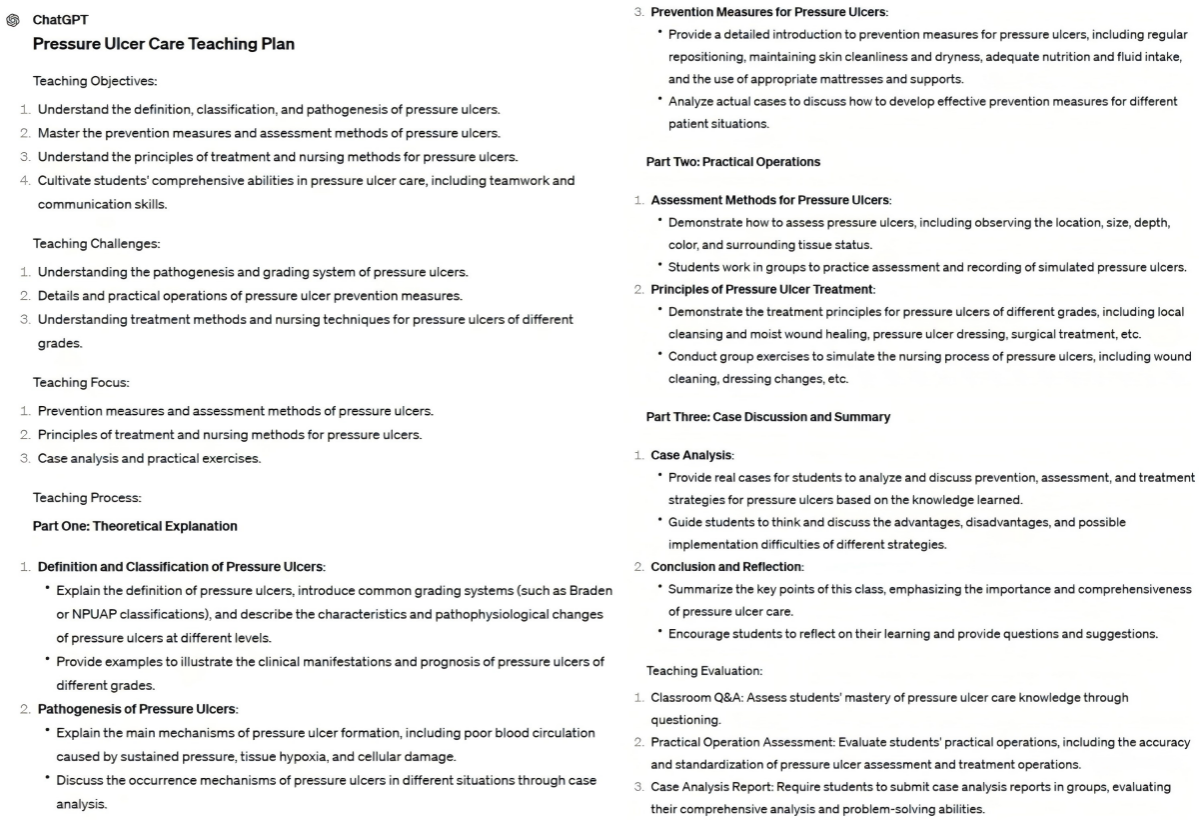
Lesson plan for pressure ulcer care.

ChatGPT is crucial in educational curriculum development since it helps teachers enhance their logical thinking and task performance skills [17]. It assists teachers create lesson plans, course handouts, and lesson plan content, which accelerates curriculum development [13]. For example, we asked ChatGPT to create a teaching plan on pressure ulcer care, and the resulting content is presented in Figure 4. Upon assessing the information, it is obvious that ChatGPT could generate core teaching plan content. However, teachers must supplement with more precise and detailed knowledge points, as well as validate the created information.

Furthermore, ChatGPT promotes innovation in teaching approaches by enabling scenario-based learning, role-playing, and the integration of diverse educational resources [18]. Recognizing the value of continuous pedagogical innovation, teachers are encouraged to apply the capabilities of AI, such as ChatGPT, alongside traditional teaching methods [19]. This integration allows for constant advancement in teaching approaches, which improves the effectiveness of teaching practices and enables educators to fulfill their instructional responsibilities more efficiently.

### Clinical Support

ChatGPT plays a pivotal role in clinical support, standardizing procedures, aiding in disease diagnosis, and delivering health education. In terms of literature search and operational procedures, ChatGPT is capable of accessing the most recent literature and clinical guidelines [20]. This capability enables the provision of evidence-based best practices to health care professionals, facilitating the identification of current operational procedures and the enhancement of operational protocols [21]. Regarding disease diagnosis, ChatGPT can analyze patient data and test results, assisting doctors in diagnosing conditions and offering treatment recommendations [22]. In addition, it supports health care professionals in telemedicine by engaging in real-time communication with patients and providing remote diagnosis and treatment suggestions [23]. Moreover, ChatGPT serves as a valuable tool for patient health education. It can translate health education materials into multiple languages and deliver personalized health education using straightforward language, thereby aiding patients in understanding condition guidance and adopting healthier lifestyles [24].

Overall, ChatGPT enhances medical education by integrating essential functionalities into clinical support through standardized processes, aiding in disease diagnosis, and delivering health education [25]. This not only empowers health care professionals to provide medical services more efficiently but also provides students with a comprehensive and enriching learning experience, fostering the growth of medical professionals with practical skills and professional competence.

### Disciplinary Development

The integration of AI and medicine represents a future frontier, characterized by the convergence of technological innovation and advancements in medical care [26]. This cross-disciplinary collaboration not only promotes technical advancement but also drives medical treatment to better intelligence and efficiency. Furthermore, it promotes the development of comprehensive capabilities in academic institutions that can navigate the junction of medicine and technology. In the future, coordinated activities in medical and technical domains have the potential to expedite technological innovation and move the field of medicine forward. This collaboration promises to usher in a new era of health care marked by innovation and efficiency, efficiently meeting society’s changing health needs [27]. ChatGPT advances medical education by enabling interdisciplinary collaboration and encouraging innovation at the confluence of medicine and technology.

## Challenges

### Learning Dependency and Uneven Education

While ChatGPT can serve as a valuable learning aid by providing answers, assisting in understanding complex concepts, and offering personalized tutoring, excessive reliance on it can yield detrimental consequences in the long term [10]. Overdependence on ChatGPT may result in the erosion of critical thinking skills, creativity, and self-directed learning capabilities. The ease of obtaining answers quickly through ChatGPT may foster complacency among students, discouraging them from engaging in reflective problem-solving [28]. To mitigate this issue, students should proactively disclose which parts of their work were aided by ChatGPT, allowing teachers to assess the overall quality of assignments more accurately [10]. Failure to do so may lead to the perpetuation of an “information cocoon,” wherein students are only exposed to solutions that align with their existing preferences, hindering the exploration of diverse perspectives. Furthermore, the widespread adoption of ChatGPT may exacerbate educational inequalities. Developing and underdeveloped countries may lack the necessary technological infrastructure and resources to fully leverage ChatGPT, widening the educational gap between these regions and more developed countries [29]. Hence, it is crucial to address these challenges to ensure equitable access to educational resources and opportunities worldwide.

### Copying and Plagiarism

When tackling assignments or final papers, students often seek to leverage technology to address challenges, enhance content, and elevate the overall quality of their work. ChatGPT, with its exceptional social capabilities and vast knowledge base, offers students the opportunity to obtain answers, prepare for exams, outline papers, and even complete them through mutual dialogue [29]. However, such practices are commonly perceived as copying answers and plagiarism, thereby contravening the fundamental principles of scientific research and academic integrity [30]. Moreover, ChatGPT encounters issues related to the fabrication of reference citations, as it may generate citations without verifiable sources, making it difficult for users to locate the original literature on academic platforms [31]. This inability to access and verify the sources provided by ChatGPT poses significant obstacles to maintaining academic integrity and conducting rigorous scholarly research [32].

It is noteworthy that Som Biswas, a radiologist in the United States, has authored 16 papers with ChatGPT, resulting in the publication of 6 articles across 4 journals [33-38]. Nevertheless, a thorough examination of the content by experts in the field has revealed significant inaccuracies, with all references found to be fictitious [39]. The editors of the journal Nature stated that while ChatGPT cannot be held accountable for the content and integrity of scientific studies, its contributions can be recognized [40]. Similarly, scientific journal editors claimed that using ChatGPT-generated content without adequate citations may be considered plagiarism, while contributions to ChatGPT might be acknowledged in the acknowledgments section [40]. Therefore, there is an urgent need to develop clearer criteria for distinguishing between authorized use and plagiarism when using ChatGPT assistance in academic research.

### Insufficient Factualness, Timeliness, and Interpretability

ChatGPT’s credibility is not absolute, as it grapples with issues, such as illusion, poor timeliness, and interpretability, akin to other large language models [41]. Despite its impressive performance, ChatGPT has been known to generate convincing yet erroneous information, undermining its reliability, particularly in the health care domain. Furthermore, because its training data is only up to January 2022, its conclusions may not always be up to date [12]. Furthermore, ChatGPT lacks specialist medical knowledge and may struggle to understand complicated illness relationships [42]. Its algorithms function as a black box, providing findings without divulging the underlying mechanism, creating ambiguity about their applicability for health care applications [43]. While doctors may leverage ChatGPT for disease diagnosis to enhance clinical accuracy, the absence of evidence supporting diagnosis and treatment may leave patients questioning the reliability of the results.

### Ethical Issues

While ChatGPT has the potential to revolutionize medical education, it also raises a number of ethical concerns. It is of the utmost importance to address issues, such as algorithmic discrimination, the allocation of responsibility for medical malpractice involving ChatGPT, and the safeguarding of data privacy and security [44,45].

ChatGPT displays algorithmic biases, including gender stereotypes, racial discrimination, and cultural insensitivity [46]. These biases not only undermine the model’s accuracy, fairness, and reliability, but they also perpetuate disparities in clinical health care [29]. Furthermore, ChatGPT’s inadequacies, such as inadequate timeliness, interpretability, and accuracy, increase the risk of incorrect clinical diagnoses, treatment protocols, and the transmission of incorrect information, risking patient care [47]. Furthermore, safeguarding patient privacy is critical in the therapeutic arena, mandating strict safeguards for sensitive patient data. Given that ChatGPT may share information, there may be a possibility of privacy breaches, as patient data are temporarily stored on Open AI’s servers [48]. This raises concerns regarding the potential leaking of patients’ private information, such as personal details, medical conditions, and examination results, while using ChatGPT for assisted diagnosis, treatment, and health education [49]. Hence, there is an urgent need to establish practical ethical norms to harness the value of ChatGPT while ensuring alignment with scientific and technological advancements and societal development goals. These norms should address concerns related to algorithmic biases, data privacy, accuracy, and accountability to foster responsible and ethical use of ChatGPT in health care and other domains [50].

### Undermining Communication and Trust

Currently, research on the applications of ChatGPT for clinical communication and health education is limited, and the outcomes are not substantial [51]. Health care professionals’ humanistic care qualities, as well as the emotional rapport they build with patients, are critical components of disease treatment [49]. While ChatGPT can aid in accomplishing clinical tasks, it lacks emotion, empathy, and the capacity to perceive patient emotions. Therefore, health care professionals cannot depend on ChatGPT for communication and health education with patients [52]. This necessitates health care professionals integrating emotional value into patient interactions, fostering a more holistic approach to care, and combining the rationality of AI with the empathetic senses of health care providers. By striking a balance between technological assistance and human compassion, health care professionals can cultivate a patient-centric environment that addresses both medical needs and emotional well-being.

## Strategies

Table 1 summarizes the challenges and strategies of ChatGPT.

**Table 1:** The challenges and strategies of ChatGPT.

Challenges	Strategies	Feasible plans
Learning dependency and uneven education	Delineate the relationship between individuals and ChatGPT Apprise the limitations of ChatGPT Optimize curriculum design	ChatGPT serves as a useful tool to aid us Adopt an objective and cautious stance Integrate ChatGPT into medical curriculum Optimize methods for assessing assignments and grades
Copying and plagiarism	Guide proper use Evaluate and review the content Develop guidelines and regulatory mechanisms	Address the basic principles and ethical boundaries and enhance the awareness of academic ethics and integrity Develop specialized software or use fake text detection technology Formulate guidelines and management mechanism
Insufficient factualness, timeliness, and interpretability	Enhance and tailor training methodologies Improve accuracy Improve transparency and interpretability	Large-scale training and use transfer learning Fine-tuning and reinforcement learning Establish visual interfaces, output human-machine interaction code, and using XAI^a^ techniques
Ethical issues, algorithmic discrimination, responsibility allocation, and safeguarding data privacy and security	Address algorithmic discrimination Assign responsibility Protecting personal privacy and information data	Large-scale training, XAI, and quality improvement Review content, involve health care professionals in decision-making, and ensure patients’ right Conceal original motives, implement manual review mechanisms, data encryption, anonymization, and establish guidelines, ethical frameworks, and institutional norms
Undermine communication and trust	Humanistic care and emotional support from health care professionals Teacher’s quality education	Provide emotional support and humanistic care Implement student-centered education and cultivate excellent qualities

^a^XAI: explainable artificial intelligence**.**

### Prevention of Overdependence

#### Delineate the Relationship Between Individuals and ChatGPT

Humans remain the primary agents in social activities, with ChatGPT serving as a useful tool to aid them [53]. While students may use the answers and suggestions provided by ChatGPT, it is crucial that they engage in critical thinking and judgment to arrive at their conclusions [54].

#### Apprise the Limitations of ChatGPT

Despite students’ optimism toward ChatGPT [55], educators must underscore that it is not a panacea and guide students to adopt an objective and cautious stance [30]. Educators can focus on nurturing students’ independent thinking, creative problem-solving abilities, and information literacy skills. This includes cultivating habits of reading and lifelong learning, fostering critical thinking and effective communication, and enhancing independent problem-solving skills [21]. The ultimate goal is to empower students to transition from mere questioners to creators and decision makers [56].

#### Optimize Curriculum Design

This can include introducing a teacher-student-machine interaction paradigm for instruction, giving courses on AI, and experimenting with new assignment forms and evaluation methodologies [57].

1. Integrating ChatGPT into the medical curriculum: problem-based learning allows students to analyze clinical problems, encouraging proactive thinking, and comparing their own ideas to those generated by ChatGPT [58]. This comparison encourages students to reflect on the strengths and limitations of both human-generated and AI-generated solutions, fostering a deeper understanding of clinical reasoning and decision-making processes. In addition to integrating ChatGPT with problem-based learning, it can also be combined with other instructional methods, such as case-based learning, team-based learning, group meetings, etc [59]. By using diverse teaching modalities, critical thinking and innovative thinking among students can be nurtured.
2. Update the methods for assessing assignments and grades. Educators might replace typical writing tasks with presentation reports, oral debates, group discussions, and peer reviews [17]. Encouraging students to preserve transcripts of their interactions using ChatGPT can also help [31]. Rather than relying on ChatGPT for direct responses, emphasizing knowledge and competency allows students to interact more deeply and think critically.

### Academic Integrity

#### Guide Proper Use

ChatGPT has distinct advantages, and it is critical not to restrict students from using it totally, but rather to guide them to use right [60]. Thus, improving the quality of student learning is critical. In terms of educational orientation, teachers ought to lead students toward developing appropriate values about science and technology. Prioritizing education on academic integrity is crucial, with a focus on reiterating the basic principles and ethical boundaries of scientific research, enhancing awareness of academic ethics and integrity, and deepening reverence for science. In addition, organizations and institutions can conduct academic integrity seminars to educate individuals about the ethical use of ChatGPT [57]. Educators should also educate students about the consequences and repercussions of violating research integrity.

#### Evaluate and Review the Content

The text produced by ChatGPT should be evaluated and reviewed to ensure academic accuracy and integrity [61]. Developing specialized software or using fake text detection technologies created particularly to recognize text generated by ChatGPT can help detect whether a communication contains faked or nonsensical text [62]. To ensure that the answers are correct, they must be approached with reasonable skepticism and verified for accuracy. It is also vital to clearly identify which pieces came from ChatGPT. For example, using plagiarism detection software allows students to ensure that the information generated by ChatGPT does not infringe on other people’s academic work, lowering the danger of plagiarism and ensuring the accuracy and authenticity of cited references [63].

#### Develop Guidelines and Regulatory Mechanisms

Stakeholders can collaborate to create relevant recommendations for the standardized use of ChatGPT [64]. Simultaneously, implementing a corresponding management system can enhance the management approach by providing training, education, assessment, review, feedback, and improvement activities to ensure the ethical use of ChatGPT [65].

### Model Enhancement

#### Enhance and Tailor Training Methodologies

While ChatGPT boasts significant power, its susceptibility to hallucination poses a challenge to its credibility. However, effective mitigation of this issue and refinement of its specialization could unlock limitless potential in the medical domain [66]. Using regular input of data into the model or using transfer learning can effectively augment a vast, diverse, accurate, and high-quality training dataset, thereby enhancing the performance of ChatGPT [67].

#### Improve Accuracy

Through fine-tuning or reinforcement learning, the process of continuously incorporating user feedback, collecting and analyzing suggestions, and reintegrating ChatGPT resources is achieved to sustainably improve ChatGPT performance [62]. Collecting feedback on ChatGPT responses allows for iterative adjustments and enhancements to the model’s accuracy [68]. Timely updates of data, especially for time-sensitive matters, facilitate a more accurate understanding of queries and the generation of relevant answers. For instance, regular updates on clinical data, research findings, expert consensus, and medical guidelines can effectively inform clinical practice.

#### Improve Transparency and Interpretability

ChatGPT's transparency can be improved by creating visual interfaces, generating human-machine interaction code, and using explainable AI techniques [8]. Increased openness builds confidence between humans and robots. Not only does it help medical workers understand the model’s decision-making process, but it also allows for improved evaluation and interpretation of generated outcomes, lowering the risk of medical errors [69]. Incorporating transparency measures is thus critical for increasing ChatGPT’s use in medical settings.

### Emphasizing Ethical Issues

Value alignment is a contentious subject in ChatGPT, with the goal of aligning its capabilities and actions with human objectives, ethical standards, and values in order to promote safety and confidence in human-ChatGPT cooperation. The top 3 ethical concerns related to ChatGPT include algorithmic discrimination, medical liability allocation, and privacy and security difficulties [49].

#### Address Algorithmic Discrimination

To address algorithmic bias, on the one hand, incorporating diverse and balanced samples for large-scale training can enhance ChatGPT’s fairness awareness [70]. On the other hand, using explainable AI can help identify biased patterns in ChatGPT while implementing fair models [8]. Furthermore, continuous review and quality improvement can minimize gender and racial discrimination in the health care sector to the greatest extent possible [44].

#### Assign Responsibility

Clear responsibility allocation is paramount when collaborating with ChatGPT for clinical disease diagnosis and health education. In other words, ensuring maximum safety in the clinical use of ChatGPT requires explicit delineation of responsibilities. Health care professionals using ChatGPT for decision support should actively engage in its decision-making process and critically assess its recommendations. Collaboration between health care professionals and ChatGPT can enhance decision-making accuracy [71]. Patients’ right to information should be upheld. Organizations and institutions must ensure ChatGPT’s responsible participation in treatment and adherence to ethical standards [72].

#### Protecting Personal Privacy and Information Data

Measures such as analogously asking ChatGPT questions to conceal original motives, implementing manual review mechanisms for uploaded information, data encryption, and anonymization can mitigate privacy risks [73]. Compliance with privacy laws and regulations is essential to secure patient privacy and medical information, ensuring data handling and storage integrity [43]. Establishing clear guidelines, ethical frameworks, and institutional norms for data collection, storage, and use is crucial [66].

### Irreplaceability of Medical Personnel and Educators

#### Humanistic Care and Emotional Support From Health Care Professionals

While ChatGPT has the potential to enhance access to primary health care in underdeveloped regions and streamline repetitive tasks for medical staff, it cannot be considered a substitute for health care professionals in any capacity [13]. ChatGPT is devoid of the attributes that are typically associated with independent consciousness, ethical standards, emotional empathy, and the capacity to anticipate unforeseen circumstances. The interpersonal communication between health care professionals and patients, as well as the emotional support and humanistic care that are provided in person, cannot be replicated by ChatGPT [74]. Furthermore, medical professionals provide invaluable empirical assistance and support to patients based on their clinical experience, which enhances the quality of clinical services. This is a capability that ChatGPT does not possess [74]. Consequently, ChatGPT should be regarded as a valuable adjunct to the work of medical personnel in clinical settings, rather than as a replacement for human health care providers.

#### Teacher’s Quality Education

Education fosters individual growth, community advancement, and the continuation of human civilization. While ChatGPT has effectively helped to progress education by equalizing and enriching instructional resources, it is still only a tool for teachers, and not a replacement [75]. Teachers have distinct powers that ChatGPT lacks. They practice student-centered teaching and help kids develop moral qualities and abilities like ideals, beliefs, values, critical thinking, emotional intelligence, and creativity. Furthermore, people are naturally social animals that require interpersonal interactions to thrive and find spiritual fulfillment.

In conclusion, despite the benefits of ChatGPT, technology cannot replace humans in health care and education. Our individual features and capacities allow us to maintain social value in an era of rapid developments in AI.

## Conclusion

ChatGPT has demonstrated considerable potential in medical education, but it has also introduced a number of thought-provoking difficulties. As ChatGPT advances, it may present new obstacles and opportunities. On a worldwide basis, cultural diversity is under peril. ChatGPT disseminates information based on the training data it gets, making well-known and popular information more easily shared and transferred. However, this has the tendency to marginalize niche or local cultures and languages, hence reducing cultural variety. Roles in the health care sector may change. The increased implementation of automation and intelligent technologies may result in the displacement of positions, such as primary diagnostic personnel and imaging diagnostics. But this might also open new career paths for professionals with expertise in medical AI and information management. Promoting AI technology development in developing nations can help advance fields, such as the creation of diagnostic software.

It is imperative that we approach ChatGPT with caution and subject it to critical evaluation, weighing its benefits against its drawbacks. This paper presents a dialectical examination of the current state of ChatGPT application in medical education, conducting an in-depth analysis of its advantages and the dilemmas it presents. In addition, targeted strategies are proposed to address these challenges effectively. The aim is to standardize and rationalize ChatGPT’s maximum potential in the future, paving the way for innovative approaches in medical education and contributing to the advancement of medicine.

## Abbreviations

AI: artificial intelligence
